# Evaluation and consequences of heterogeneity in the circulating tumor cell compartment

**DOI:** 10.18632/oncotarget.8015

**Published:** 2016-03-09

**Authors:** Anja Brouwer, Bram De Laere, Dieter Peeters, Marc Peeters, Roberto Salgado, Luc Dirix, Steven Van Laere

**Affiliations:** ^1^ Center for Oncological Research (CORE), University of Antwerp, Antwerp, Belgium; ^2^ Department of Oncology, Antwerp University Hospital, Antwerp, Belgium; ^3^ Department of Pathology, GZA Hospitals Sint-Augustinus, Antwerp, Belgium; ^4^ Department of Oncology, GZA Hospitals Sint-Augustinus, Antwerp, Belgium; ^5^ Breast Cancer Translational Research Laboratory, Jules Bordet Institute, Brussels, Belgium

**Keywords:** circulating tumor cells, heterogeneity, liquid biopsy

## Abstract

A growing understanding of the molecular biology of cancer and the identification of specific aberrations driving cancer evolution have led to the development of various targeted agents. Therapeutic decisions concerning these drugs are often guided by single biopsies of the primary tumor. Yet, it is well known that tumors can exhibit significant heterogeneity and change over time as a result of selective pressure. Circulating tumor cells (CTCs) are shed from various tumor sites and are thought to represent the molecular landscape of a patient's overall tumor burden. Moreover, a minimal-invasive liquid biopsy facilitates monitoring of clonal evolution during therapy pressure and disease progression in real-time. While more information becomes available regarding heterogeneity among CTCs, comparison between these studies is needed. In this review, we focus on the genomic and transcriptional heterogeneity found in the CTC compartment, and its significance for clinical decision making.

## INTRODUCTION

Metastatic disease is responsible for over 90% of cancer-related deaths [1]. Due to a growing insight in the molecular mechanisms driving cancer evolution and identification of specific molecular aberrations involved, an increasing number of patients is now considered candidate for treatment with so called targeted agents [2, 3]. However, when it comes to therapy decision making, intra-patient heterogeneity should be taken into account. Here we discuss the molecular heterogeneity within the circulating tumor cell (CTC) compartment in various tumor types. Furthermore, we review the causes and consequences of this heterogeneity and the clinical perspective.

### Intra-tumor heterogeneity

Advances in DNA sequencing techniques and comparison of tumor samples obtained from different sites and at different time points, have revealed an extensive view on clonal evolution and intra tumor heterogeneity (ITH). During tumor development, cancer cells acquire various aberrations, including both passenger (neutral) and driver (advantageous) mutations. Due to selection and clonal expansion, multiple genetically distinct subclones can emerge that often evolve following a pattern of branched evolution, which has been described for various solid tumor types [4–15]. This branched evolution comprises multiple subclones that have a phenotypic advantage within a particular environment and evolve simultaneously resulting in ITH, whereas a linear evolutionary pattern describes a random genetic drift where fitter clones outgrow ancestral clones, resulting in a relatively homogeneous tumor at any given moment [16]. Exome sequencing of multiple tumor foci from clear-cell renal carcinomas revealed that only one-third of the identified driver aberrations were present in every region analyzed from an individual tumor, suggesting these to be early founder aberrations. In contrast, 71% of driver mutations were heterogeneous between tumor regions, although appearing clonally dominant within individual regions, showing branched evolution with spatially separated dominant subclones [6].

During the development of metastatic disease, tumor cells shed from the primary tumor are able to travel to distant organ sites to seed metastatic tumors [17]. Moreover, in breast, prostate, and pancreatic cancers, it has been shown that these cells disseminate long before metastatic colonization becomes clinical evident [18, 19]. Both early and late dissemination, as well as polyclonal and bidirectional seeding between different tumor sites, and parallel evolution have been described [20, 21]. Hence, different tumor sites will consist of unique evolutionary landscapes, leading to inter-metastasis heterogeneity [12, 21–23].

Although clonal diversity can be resolved by spatial sampling [7] in combination with deep-sequencing of tumor tissue to determine (sub)clonality of certain mutations [9–11, 24, 25], the field is shifting towards single cell sequencing (SCS) studies to shed light on this heterogeneity. SCS allows to study rare tumor cell populations and clonal expansion, and is already widely used in hematopoietic cancers, including Acute Myeloid Leukemia [26, 27]. However, in solid tumors, patients often exhibit multiple lesions composed of genetically diverse subclones that evolve in parallel over time [28, 29], hampering the evaluation of targetable aberrations in a patient's metastatic disease [22, 30, 31]. Hence, single tumor biopsies fail to represent the clonal landscape of the overall tumor burden. Moreover, changing biology and resistance patterns, influenced by prior therapies, stresses the need for repeated sampling of a patients tumor burden, to expose the molecular landscape at various moments in time [23, 32].

### Circulating tumor cells

CTCs are shed into the peripheral blood from various tumor deposits and represent the actual tumor mass as was demonstrated by comparative analysis of CTCs, primary tumors, and metastases in various tumor types [33–37]. CTC capturing systems have revealed that aggressive tumors release thousands of cancer cells into the circulation each day [38–41], although most CTCs only persist for a short time in the circulation, with an estimated half-life between 1 and 24 hours [38, 42, 43]. It is assumed, however, that CTCs with an intermediate phenotype between epithelial and mesenchymal have the highest plasticity and can survive in the circulation [44–46]. Although CTCs are a frequent phenomenon in cancer, only a small fraction (< 0.01%) eventually succeed in forming metastasis [47, 48]. This was further demonstrated with the identification of specific subsets of CTCs with tumor-initiating capacity [39, 40, 49, 50].

In general, CTCs are relatively rare, representing only one in more than a million blood cells [40]. Still, CTC count of patients with metastatic cancer is a strong prognostic factor for overall survival in several tumor types [51–60]. Moreover, changes in CTC counts during treatment are used as a marker for therapy response [42, 55, 61–64]. Genotyping of circulating tumor (ct)DNA, derived from tumor deposits and lysed CTCs, also has the potential to serve as a marker for tumor burden, therapy response, and even therapy resistance patterns, when followed longitudinally [32, 65–68]. Moreover, mutation levels in plasma can reflect the multifocal clonal hierarchy of tissue biopsies from a patient with metastatic breast cancer during therapy [23]. Compared to CTCs, ctDNA is easier and less laborious to obtain. Nonetheless, CTCs represent pure and intact tumor cells. Molecular analysis on DNA, RNA, and protein level [33, 69], as well as functional cellular characteristics can only be interrogated in CTCs [39]. In addition, molecular analysis of CTCs enables researchers to detect the presence of multiple mutations within the same cell, in order to decipher tumor heterogeneity and map clonal evolution. When combining genomic and transcriptomic evaluation of CTCs, a potential linkage between mutational status and pathway activation can be observed [70].

CTCs can be analyzed both as pure cells as well as enriched fractions. Mutation detection of DNA extracted from CTC-enriched samples demonstrated activating mutations in the *EGFR*, *KRAS*, and *AR* genes in patients suffering from lung cancer, colorectal cancer (CRC), and castration-resistant prostate cancer (CRPC) respectively [65, 71, 72]. Additionally, RNA analysis of enriched CTC fractions have been performed using reverse transcription PCR (RT-PCR) amplification of tumor-specific transcripts, such as AR splice variant 7 in CRPC, and translocations like *EML4–ALK* in lung cancer and *TMPRSS2–ERG* in prostate cancer [42, 73–75]. However, sequencing of enriched fractions iscomplicated by low levels of tumor-specific templates and contamination by abundant leukocyte-derived sequences, limiting the sensitivity and specificity [76, 77]. Advances in next generation sequencing (NGS) strategies and computational analyses help resolve this challenge. Nevertheless, single CTC sequencing strategies can provide a direct insight into CTC heterogeneity by identifying co-existing mutations within a cell. Heitzer and colleagues, profiled individual CTCs isolated from patients with metastatic CRC, using array-Comparative Genomic Hybridization (CGH) and targeted panel sequencing of 68 genes. Various genomic aberrations in CTCs were found, indicative for their subclonal origin from specific areas of the original tumor [33].

**Figure 1:**
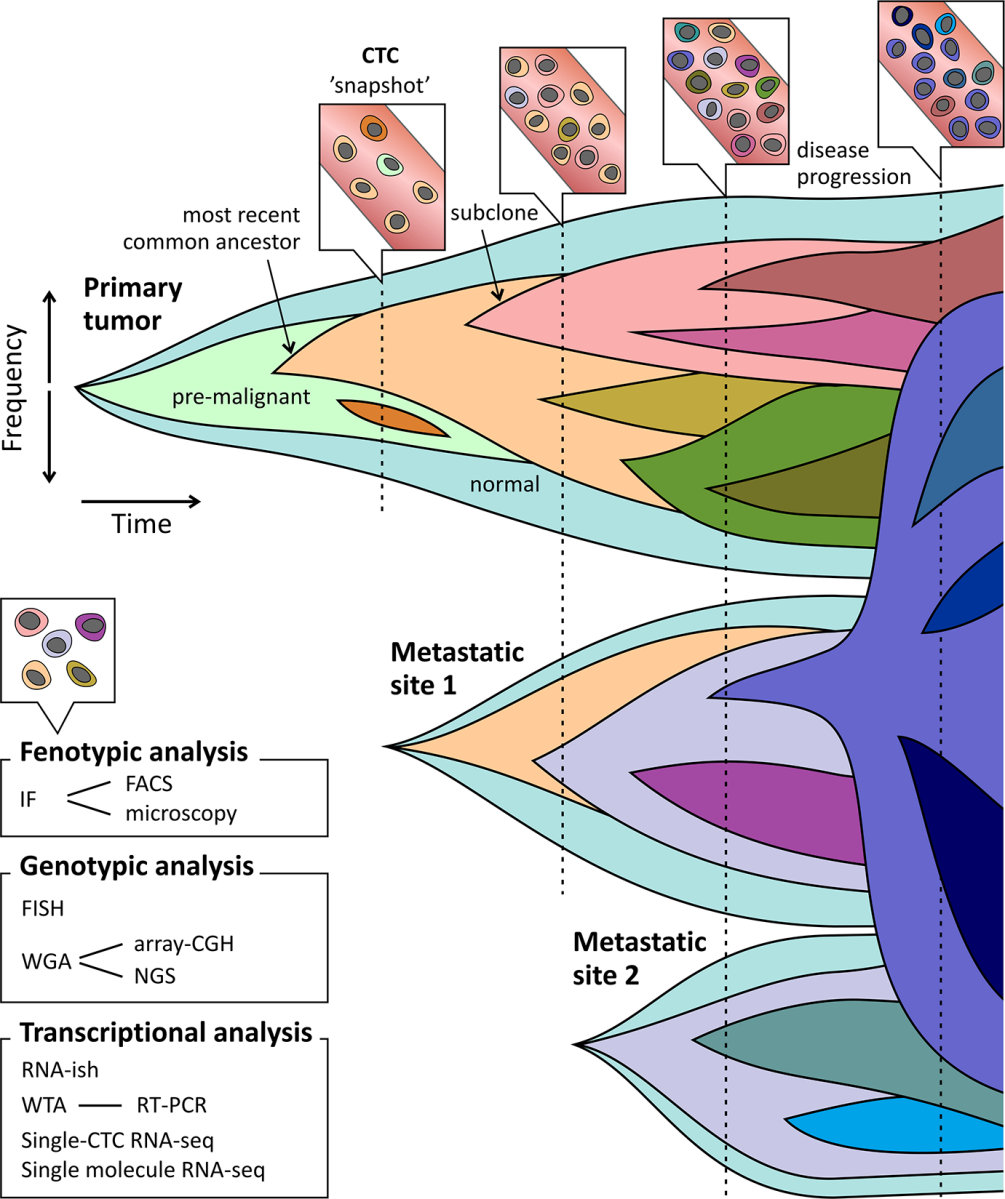
CTCs as snapshot of the evolving tumor landscape Clonal evolution depicted as emergence of clones after acquisition of driver mutations. New (sub) clones derive from ancestral clones following linear and branched evolution. Outgrowth and repression (therapeutic or outcompeting) of these subclones can lead to emergence and disappearance of driver mutations respectively. Seeding and re-seeding of tumor cells causes development of changing tumor landscapes at multiple sites. Selective therapy pressure can lead to outgrowth of resistant clones at time of disease progression. CTCs sampling can function as a snapshot of the overall tumor bulk (primary tumor and metastases). When profiling CTCs at multiple time points emerging and decreasing subclones can be unveiled. Techniques to profile CTCs include phenotypical and molecular analyses.

Overall, cancer presents a problem of continuous spatial and temporal complexity, particularly due to selection pressures such as anti-cancer drugs, that may promote dominance of previously minor or dormant lineages [78]. It is important to note that subclonal diversity is viewed as a snapshot, and only serial analysis of CTCs can clarify the much needed dynamic view of tumor genomes, as pointed out in Figure 1. Both in metastasis research, as well as in clinical practice, it is important to know whether a minor subclone is emerging or has been outcompeted by the dominant subclone [16]. Longitudinal CTC studies have been performed to investigate the clonal changes in both phenotypical and molecular profiles associated with disease evolution and therapy resistance [79–81]. Hence, CTCs might reflect the characteristics of the current status of the biologically and clinically relevant subclones irrespective of a detailed anatomical distribution, and should ideally be suited to provide dynamic assessments of tumor characteristics in patients with metastatic disease. Even more since repeated sampling of multiple metastatic lesions is an invasive procedure and often not feasible.

Although increasingly sophisticated technologies have become available to detect and isolate CTCs, as is already extensively reviewed [82–88], further progress in CTC research is needed to envision heterogeneity and clonal evolution within the CTC compartment. Major questions in CTC research implicate the clonal relationship between CTCs and the number of CTCs that have to be analyzed in order to capture the overall profile of the dominant disease driving (sub)clones in a patient suffering from widespread metastatic disease. In this review, we will focus on the genomic and transcriptional heterogeneity found in the CTC compartment, and its significance for clinical decision making.

## GENOTYPIC CTC HETEROGENEITY

A growing number of research articles have been published demonstrating genotypic heterogeneity in the circulating compartment, emphasizing the need for studies analyzing multiple purified CTC samples. This can be performed focusing on several types of aberrations such as gene rearrangements, mutations, and CNA profiles. Here we compare the results regarding genomic variation in CTCs of various tumor types (summarized in Table 1). We found that in many patients rearrangements as well as specific and global mutation profiles were highly heterogeneous. Concerning CNA profiles, homogeneity in overall profiles was reported frequently, although in both breast and prostate cancer intra-patient variation was observed. Furthermore, changes in CNA profiles over time were documented and in depth analysis of copy number profiles of specific genes in various tumor types demonstrated extensive heterogeneity.

**Table 1:** Genomic heterogeneity in CTCs

#CTC	#Pts	Isolation	Analysis	Targets	Heterogeneity	Ref.
*Lung cancer*						
n.s.	32	MF (ISET)	FA-FISH	*ALK* rearrangements	18 ALK+ patients exhibited between 7 and 24 CTCs/ml, mean percentage of *ALK*-rearranged CTCs was 63% (range 28-100%). All ALK– patients had < 4 rearranged CTCs.	89
n.s.	5	MF (ISET)	FA-FISH	*ALK* rearrangements	5 patients showed *ALK*-gene rearrangements in all CTCs (100%), while in the primary tumor only half of the tumor cells show these rearrangements.	91
177	1	microfluidics + cytospin	FISH	*ALK* rearrangements	25% of the total 177 CTC of 1 patient harbored *ALK*-gene rearrangements, and 54% of the 200 primary tumor cells did.	90
n.s.	8	MF (ISET)	FA-FISH	*ROS1* rearrangements	*ROS1* rearrangements were detected in the CTCs of all 4 ROS+ patients. *ROS1* copy number was heterogeneous within these CTCs and increased at time of disease progression.	95
8	1	CS + MM	WES	CNA; mutations; indels	CNA show inter-CTC homogeneity, and represent the metastatic tumor. SCLC and NSCLC can be differentiated based on CNA-profile. Mutations and indels were highly heterogeneous in all CTCs.	35
8 + pools	2	CS + DEPArray	WGS; TAS	CNA; *TP53, RB1* mutations	CNA strongly correlated, but 1 of 6 CTC harbored substantial CNA differences. *TP53* and *RB1* mutations were homogeneous.	112
1 pool	4	microfluidics	Allele-specific PCR	*EGFR* mutations and CNA	Temporal heterogeneity in *EGFR* mutations. Genotypes of enriched CTC fractions evolved during therapy, with consistent presence of the primary *EGFR* activating mutation and the emergence of a drug-resistant mutation.	65
*Colorectal cancer*						
37	6	CS + MM	aCGH; Panel	CNA; 68 CRC-related gene panel	Multiple CRC related CNA and mutations were found in CTC and tissue samples. Various CTC-specific mutations were detected, but retraced at subclonal level by ultra-deep sequencing of the tissue samples. Inter-CTC heterogeneity, with some private mutations.	33
741	33	CS + MM	qPCR; TAS	*EGFR* CNA; *PIK3CA, KRAS*, and *BRAF* mutations	CN-gain of *EGFR* was found in 27% of CTCs of 3 patients, 1 patient had *KRAS* mutations in 33% of CTCs, 39% of CTCs of 4 patients harbored *PIK3CA* mutations.	105
126	31	CS + MM	TAS	*TP53, KRAS* and *BRAF* mutations	CTCs were analyzed of 18 patients. 6 patients harbored heterogeneous CTC populations.	107
pools	21	DGC + DEPArray	TAS; PyroSeq	*KRAS* mutations	In 1 patient, 3 pools of CTCs had different mutational statuses, two mutations were found in the first pool and another mutation in a second pool of isolated CTCs.	108
pools	2	CS enriched	qPCR	*KRAS* mutations	Temporal heterogeneity: enriched CTC fractions exhibited different mutational status of KRAS during treatment.	109
*Prostate cancer*						
n.s.	49	CS	On-chip FISH	*ERG* rearrangements;*PTEN* and *AR* CNA	FISH on CTCs revealed homogenous *ERG* rearrangements but heterogeneous *AR* amplifications and *PTEN* deletions.	96
n.s.	77	CS + cytospin	FISH	*AR* and *MYC* CNA	There was considerable variability in the morphology of CTCs in individual patients. 1 patient showed heterogeneity of FISH patterns, with *AR* amplification in a subset of CTCs, but all with high copy number gain for *MYC*.	117
n.s.	7	DGC + cytospin	FISH	*BRCA1* CNA	In 4 of 7 patients, *BRCA1* losses appeared in a fraction of CTCs.	118
pools	9	IE/FACS	aCGH	CNA	CTCs from all patients revealed a wide range of CNA. Replicate CTC isolates where comparable showing gains in the *CCND1* and *AR* locus.	114
41	1	HD-CTC + MM	WGS	CNA	Three different clonal lineages were found. Clone B was present subclonally at first blood draw, but demonstrated outgrowth in the third blood draw. A third clone emerged at fourth blood draw.	80
19 + 10	2	MagSweeper + MM	WES	Somatic SNV	Although non-uniform coverage, a heterogeneous mutation profile was detected in single CTCs. When pooling the CTC data, found SNVs were comparable to the primary tumor.	34
*Breast cancer*						
261 + pools	42	CS + DEPArray	aCGH; qPCR; TAS	CNA; *ERBB2* CNA; *PIK3CA* mutations	2 patients had heterogeneous *PIK3CA* mutational status in their single and pooled CTCs. 10 of 16 patients harboring *PIK3CA* mutations showed molecular heterogeneity based on CNA. *ERBB2* amplification was uniformly detected in all CTCs of 7 patients.	101
26	12	CS + flow sorting (MoFlo XDP)	aCGH; qPCR; TAS	CNA; *CCND1* CNA; *PIK3CA* mutations	CNA were found breast cancer related in all CTCs, but differences in CNA between related CTCs were present in all cases. 1 patient harbored a mutation in exon 20 of the *PIK3CA* gene in both CTCs and 1 patient harbored another *PIK3CA* mutation in 1 of 1 CTCs.	100
147 + pools	26	CS + DEPArray	TAS	*PIK3CA* hotspots	11 of 26 patients were found to harbor a heterogeneous *PIK3CA* mutational status in their CTC compartment.	102
115 + pools	18	CS + DEPArray	TAS	*PIK3CA* hotspots	3 patients were homogeneously mutated in all CTCs. 1 patient was found to have three different *PIK3CA* mutations.	104
185	17	MagSweeper + MM	TAS	*PIK3CA* hotspots	1 patient harbored a heterogeneous CTC compartment based on *PIK3CA* status.	103
11 + pools	2	CS + DEPArray	TAS	*TP53* mutations	In one patient, 2 of 6 single CTC harbored two different *TP53* mutations. In the second patient, 3 of 5 single and 5 of 6 clusters of CTCs showed a *TP53*^R110delC^ mutation.	111
402	3	DGC + cytospin	IF/FISH (BioView)	*EGFR* CNA	10 of 91 ALDH1+/HPSE+ cells showed *EGFR* amplification. This was 19 of 311 in the ALDH1–/HPSE+ population.	50
31 + pools	1	CS ór DGC + MM	WGS; aCGH	CNA	CNA show homogeneity within all isolated CTCs.	36
n.s.	3	IE/FACS	aCGH	CNA	Temporal heterogeneity: Serial testing of enriched CTC populations revealed numerous additional CNA beyond the baseline profile.	116
*Melanoma*						
24 + 18	2	Microfluidic + LCM	TAS	*BRAF* mutations	Consistency in the *BRAF*^V600E^ mutation, and in accordance with the primary tumor.	110
15	7	IM + MM	CGH	CNA	In 5 of 6 patients with ≥ 1 isolated CTC, hierarchical clustering showed a clonal origin.	115
*Multiple cancers*						
n.s.	20	IM + cytospin	FISH	CNA	6 patients had a homogeneous pattern of aneusomy in all CTCs. In 10 patients a heterogeneous pattern was observed, including 6 cases with two distinct clones.	49

**Abbreviations:** aCGH, array comparative genomic hybridization; CNA, copy number alterations; CS, CellSearch enrichment; CTC, circulating tumor cell; DGC, density gradient centrifugation; FA-FISH, filter adapted fluorescent *in situ* hybridization; HD-CTC, high-definition CTC assay; IE/FACS, immunomagnetic enrichment and fluorescence-activated cell sorting; IF, immunofluorescence; IM, immunomagnetic enrichment; LCM, laser capture microscopy; MF, microfiltration; MM, micromanipulation; TAS, targeted amplicon sequencing; qPCR, quantitative polyclonal chain reaction; WES, whole exome sequencing; WGS, whole genome sequencing; n.s., not specified.

### Rearrangements

Several research groups studied rearrangements of the *ALK* gene in CTCs using Fluorescence *in situ* hybridization (FISH) [89–91]. In non-small cell lung cancer (NSCLC), EML4*-ALK* fusion is present in approximately 3–7% of cancers, and these patients are eligible for targeted treatment with crizotinib and ceritinib [92–94]. Pailler and colleagues demonstrated that percentages of *ALK*-rearranged CTCs ranged between 28% and 100% in patients with ALK-positive tumors, and varied within these patients during crizotinib therapy. This suggests that the *ALK*-rearranged CTC population might be a consequence of clonal selection from a specific subpopulation of primary tumor cells, and that outgrowth of this subpopulation can be an indication for therapy resistance [89]. Percentages of *ALK*-rearranged CTCs were confirmed by two other studies. In a first report, one-fourth of the total 177 CTCs of one patient harbored *ALK* rearrangements [90] and in the other, 100% of the CTCs of 5 patients were *ALK*-rearranged [91], whereas in the primary tumor tissue this was around 50% in both studies. Furthermore, *ROS1* rearrangements were found in CTCs of four patients with lung cancer [95]. FISH has also been used to analyze *ERG* rearrangement in prostate cancer CTCs [96, 97]. *TMPRSS2ERG* gene fusion was either homogeneously present in all CTCs of one patient or absent [96]. Although presence of this *ERG* rearrangement demonstrates a significant association with PSA response to abiraterone in this study, TMPRSS2-ERG status could not predict a decline in PSA or other clinical outcomes in response to abiraterone therapy in a clinical trial evaluating enriched CTC populations [97].

### Hotspot mutations

In breast cancer, *PIK3CA* is mutated in up to 25% of patients, with mutation frequencies rising to 40% in the hormone receptor-positive subgroups [98, 99]. Analyzing the *PIK3CA* genotype has clinical relevance with respect to drug resistance, e.g. against HER2-targeted therapy. Hence, various studies are performed investigating the *PIK3CA* mutational status in CTCs. In a first study, two single CTCs per patient were analyzed [100]. In two patients *PIK3CA* mutations were found in all CTCs of these patients (resp. 1 and 2 CTCs). In a similar, but much larger study, *PIK3CA* mutations were detected in 16 patients, two of whom harboring a heterogeneous mutational status in their single and pooled CTCs [101]. De Laere and colleagues profiled CTCs of 26 hormone receptor positive patients, ranging between 4 and 311 CTCs per patient. In 19 cases (73%) *PIK3CA* mutations were detected. Of these, six cases were found almost homogeneously mutant for one specific mutation, whereas another six patients were extensively heterogeneous with subclones harboring one or multiple *PIK3CA* mutations [102]. In contrast, another study detected *PIK3CA* mutations in only one out of 17 patients, which might be due to different patient selection [103]. Single CTCs of 24 samples (containing 2–50 CTCs) of 12 patients were examined for presence of *PIK3CA* mutations. In one patient an exon 9 mutation was detected in two out of nine serial samples, both at a heterogeneous level [103]. Pestrin *et al.* identified *PIK3CA* mutations in CTCs in 6 out of 18 patients [104]. In three cases with multiple CTCs analyzed, all CTCs were homogeneously mutant. One patient had a heterogeneous mutational status, with 3 out of 16 single CTCs harboring three different *PIK3CA* mutations [104]. When combining aforementioned studies, from a total of 47 *PIK3CA* mutated patients, 15 had a heterogeneous circulating compartment with mutated CTCs present at a subclonal level. Also in a study on CRC, *PIK3CA* mutations were present at a subclonal level in four patients; one of whom harbored two different *PIK3CA* mutations in separate CTCs [105].

Since *PIK3CA*, *BRAF*, *KRAS*, and *PTEN* are relevant genes in predicting resistance to anti-EGFR therapy [106], mutations in these genes are frequently studied using CTCs. A recent study isolated 37 single CTCs from six patients with metastatic CRC for sequencing of a 68 CRC-associated gene panel to determine mutational landscapes in CTCs and the corresponding primary tumors and metastases [33]. Point mutations in *APC, KRAS, PIK3CA*, and *TP53* in the primary tumors were also present in the single CTCs. However, 20 ‘branch’ mutations were found exclusively in CTCs, although targeted ultra-deep sequencing revealed the presence of 17 of these mutations at subclonal level in either the primary tumor or metastases [33]. Two more studies performed targeted sequencing of *BRAF*, *KRAS*, and *TP53* of respectively 741 and 126 single CTCs [105, 107]. The first study detected the presence of *KRAS* mutations in one-third of CTCs of one patient [105], while in the other, 6 out of 18 patients demonstrated a heterogeneous CTC compartment regarding these genes [107]. Moreover, two studies examined heterogeneity of *KRAS* mutations in pools of CTCs [108, 109]. Fabbri *et al.* reported one patient harboring three pools of CTCs with different mutational statuses. Two specific *KRAS* mutations were detected in the first pool, and another *KRAS* mutations was found in a second pool of pure CTCs [108]. Also, temporal heterogeneity was shown as enriched CTC fractions exhibiting different mutational status of *KRAS* during treatment [109]. However, one can argue on the sensitivity of mutation detection in enriched samples containing low CTC-counts, as often seen during therapy. Furthermore, mutational analysis was performed on multiple single CTCs collected from two patients with stage-IV melanoma. All CTCs were consistently *BRAF*^V600E^ mutated analogous to the primary tumor [110].

In a study towards *TP53* mutations, single and pooled CTCs of two patients with metastatic triple-negative inflammatory breast cancer, known for harboring a *TP53* mutation in their primary tumor, were recovered for molecular analysis [111]. In the first patient, 2 of 6 single CTC harbored two different *TP53* mutations, one of these was also found in the pool of 14 CTCs. In the second patient, 3 of 5 single and 5 of 6 clusters of CTCs had a *TP53*^R110delC^ mutation. In contrast, *TP53* and *RB1* were homogeneous in all CTCs of lung cancer patients [112].

Temporal heterogeneity was demonstrated in pools of pure CTCs from patients with NSCLC receiving tyrosine kinase inhibitors. Serial analysis showed emergence of activating mutations in the gene encoding the EGFR conferring a mechanism of acquired resistance to therapy [65]. *EGFR* mutation detection was also performed on enriched CTC samples. In 4 out 31 cases, multiple *EGFR* mutations were documented, suggesting possible CTC heterogeneity [113]. However, the actual mutational landscape and subclonality can only be detected in single CTC samples or multiple pools of pure CTCs.

### Global mutational profile

A recent study applied whole exome sequencing (WES) of 19 single CTCs from a patient with metastatic prostate cancer [34]. Although non-uniform coverage, a heterogeneous mutation profile was detected in single CTCs. To compensate for the low coverage and random polymerase errors that did occur in individual CTCs, single-CTC data was pooled. Half of the somatic SNV in CTCs could be detected in the primary and metastatic sites, whereas the rest were CTC-specific mutations [34]. Moreover, Ni *et al.* determined single nucleotide variation landscapes in CTCs of four patients with lung cancer by single-cell exome sequencing [35]. The exome data showed extensive variation from cell to cell and presence of ‘private’ CTC mutations, not detected in tissue samples. The authors raise the question of false discovery due to interfering technical errors compatible with the MALBAC method used [35].

### Copy number alterations

Methods used to study genome-wide CNA include array-CGH and whole genome or exome sequencing. In prostate cancer, a wide range of CNA in pools of pure CTCs were detected in nine patients, using array-CGH. But more specifically, CTCs showed uniform copy number gains in both the *AR* and *CCND1* locus [114]. In one study where two single breast cancer CTCs per patient were analyzed for CNA, all CTCs displayed a typical breast cancer related copy number profile [100], with six patients harboring *CCND1* amplification in both CTCs. Yet, differences in CNA between CTC couples were to a greater or lesser extent visible in all cases. Furthermore, multiple CTCs of 16 patients with breast cancer were analyzed using array-CGH. Ten of these patients showed molecular heterogeneity based on CNA. Although, in seven cases were *ERBB2* amplification was detected, it was homogeneous in all CTCs [101].

However, in multiple studies in various tumor types, homogeneity in the copy number profile was demonstrated. WES was applied to lung cancer CTCs in two studies [35, 112]. Five out of six patients had highly homogeneous copy number profiles, although one patient harbored substantial CNA heterogeneity [112]. In another study, the copy number profiles of the single CTCs were highly similar and shared most of the same CNAs as the primary and metastatic tumor cells. Furthermore, CNA patterns were indicative for specific lung cancer subtypes [35]. A recent study isolated 37 single CTCs from six patients with metastatic CRC for copy number profiling with array-CGH [33]. In general, many of the CTCs shared a number of gains and losses with the primary and metastatic lesions. However, they also observed private copy number changes in CTCs as well as heterogeneity between CTCs [33]. To define CNA in melanoma, the genomes of 15 individually isolated CTCs from seven patients were analyzed by single-cell CGH [115]. All of the analyzed CTCs displayed multiple chromosomal changes and carried aberrations typical for melanoma. In five of six cases with multiple CTCs isolated, hierarchical clustering of the CTCs showed a clonal relationship [115].

Sampling at multiple time-points to evaluate genetic evolution based on CNA profiles was performed in three studies [36, 80, 116]. Dago and colleagues thoroughly analyzed CNA of multiple single CTCs of one patient with prostate cancer by WGS at various time points. Three different clonal lineages were found. One specific clone was present at subclonal level at the first blood draw, but demonstrated outgrowth at time of the third blood draw. A third clone only emerged at the fourth time point [80]. Both array-CGH and WGS were applied for copy number analyses in one patient with breast cancer harboring extensive numbers of CTCs [36]. CNA demonstrated high similarities between the 31 single and 21 pools of CTCs ranging between 5 and 100 CTCs. Furthermore, a high degree of analogy was also found with CNA in primary and metastatic tissue samples [36]. In a large breast cancer cohort, array-CGH of CTCs revealed a wide range of CNA, including those known for breast cancer [116]. In one patient, where multiple sampling was performed, CTCs of the second blood draw revealed numerous additional CNA beyond the baseline profile, while the third sample, divided in two pools, was comparable with itself and the second. Interestingly, the patient initially responded to her cancer treatment, but subsequently developed disease progression. In two other cases temporal homogeneity was documented between first and second blood draw. Furthermore, CTCs and the primary tumor were moderately and highly correlated, respectively [116].

Then, various studies have thoroughly analyzed CNA of specific target genes using FISH. In 4 patients with lung cancer, *ROS1* copy numbers were heterogeneous between CTCs [95]. In prostate cancer, FISH was applied to study CNA of *AR*, *BRCA1*, *MYC*, and *PTEN* [96, 117, 118]. Leversha and colleagues report a considerable variability in CTCs of individual patients. In one patient, a subset of CTCs showed *AR* amplification, whereas all CTCs had high copy number gain for *MYC* [117]. A similar heterogeneity in *AR* amplifications and loss of the tumor suppressor gene *PTEN* was detected by Attard *et al.* when profiling 49 patients suffering CRPC [96]. FISH analysis further revealed *BRCA1* losses appearing in minute fractions of CTCs in four of seven patients [118]. In breast cancer, fluorescent cell sorting was combined with FISH to analyze *EGFR* amplification in CTCs [50]. 11% and 6% of CTCs from ALDH1 positive and negative populations respectively, harbored *EGFR* amplification [50]. Furthermore, *EFGR* copy number gain was found in 37% of CTCs of three patients with CRC, based on array-CGH data [105].

## TRANSCRIPTIONAL CTC HETEROGENEITY

While in diploid cells chromosomal DNA molecules are present with only two copies, a single cell harbors thousands of copies of each mRNA transcript, which facilitates single-cell RNA approaches [119]. Yet, single cell RNA studies are affected by transcriptional bursting or pulsing [120, 121]. This phenomenon can account for the high variability in gene expression between cells in isogenic populations, and therefore transcriptional heterogeneity should be evaluated with caution. On the other hand, variability in gene expression may also contribute to resistance of sub-populations of cancer cells to chemotherapy [122]. Gene-expression studies in single CTCs may be essential for determining the nature and extent of tumor heterogeneity, linking phenotypic differences with genetic and epigenetic aberrations. However, preserving RNA is more difficult than DNA and concerns have been raised about the impact of sample processing on CTC expression profiles [123]. Hence, several devices have been developed for direct and fast isolation of CTCs using a microfluidic approach [37, 75, 81, 124–126].

Single cell expression profiling is performed using RNA-*in situ* hybridization (ish), RT-PCR, and RNA-sequencing (seq). While RNA-ish has the advantage of direct analysis of the RNA without whole transcriptome amplification, expression of far more genes can be evaluated using RT-PCR or RNA-seq. Differentiating the changes in gene expression that are biologically relevant from those caused by technical and biological noise remains a significant hurdle for single-cell transcriptome studies. Hence, single cell mRNA-seq protocols are being developed with improved transcriptome coverage, high reproducibility, and low technical variation [127, 128].

Hereafter, we review various publications on transcriptional heterogeneity in CTCs. Often, patient-specific global expression profiles were observed. However, when looking in detail, significant heterogeneity between CTCs is found regarding specific transcripts, which is often linked to therapy selection or response. Table 2 gives an overview of the experimental details of these studies.

**Table 2:** Transcriptional heterogeneity in CTCs

#CTC	#Pts	Isolation	Analysis	Targets	Heterogeneity	Ref.
*Breast cancer*						
n.s.	17	^HB^CTC-Chip	RNA-ish; RNA-seq-DGE	EMT markers	Heterogeneous fractions of Epithelial (E), Mesenchymal (M), and EM-CTCs; In TNBC more homogeneous pools of M-CTCs. Temporal heterogeneity: at progressive disease, 10 patients harbored emerging numbers of M-CTCs.	81
105	35	MagSweeper + MM	qRT-PCR	87 cancer-associated genes	Two major subgroups of CTCs, i.e. high expression of EMTgenes and high metastasis-associated genes. Heterogeneity based on CTCs not clustering by patient-ID and 8 patients having CTCs in both clusters.	132
15 pools + 14 clusters	10	^CTC^-iChip + MM	RNA-Seq	Whole transcriptome	Based on global gene expression level, all isolated CTCs clustered closely by patient of origin. Based on *JUP* and 31 cluster-associated genes, CTC-clusters could be differentiated from pooled single CTCs.	124
~400	20	IM (Maintrac) + AP	PCR + gelelectro-phoresis	HER2, EpCAM, Vimentin, and NANOG	Expression patterns changed after surgery, with emerging of a sub-population of EpCAM positive CTC expressing NANOG and/or vimentin.	130
*Prostate cancer*						
77	13	^neg^CTC-iChip + MM	RNA-seq	Whole transcriptome	Single CTCs from nine individual patient with at least 3 CTCs analyzed, showed considerably higher intra-patient heterogeneity in their transcriptional profiles compared to single cells from prostate cancer cell lines.	126
20	4	MagSweeper + MM	RNA-seq	Whole transcriptome	All CTCs, except two, cluster in a patient specific manner. 181 cancer-specific genes were overexpressed in the CTCs, compared to normal tissue. Specific transcripts, e.g. related to CRPC or *ERG*-fusion, were detected homogeneously within the same patients.	131
48	2	MagSweeper + Nanowell	RNA-seq	*KLK3* (PSA) mRNA	*KLK3* expression was variable between the 26 individual CTCs, for which a sufficient number of genes including *KLK3* were covered.	34
38	8	MF + MM	qRT-PCR	84 EMT-related genes	Heterogeneous upregulation of EMT-associated gene expression, especially in CRPC.	129
pools	21	IM (AdnaTest)	qRT-PCR	AR full length + AR-V7	Temporal heterogeneity: 1 out 9 patients converted to AR-V7 positive, at progression on Taxane. While 7 out 12 patient who were at baseline AR-V7 positive became negative at progression.	73
*Pancreatic cancer*						
265	15	^HB^CTC-Chip	RNA-ish; RNA-Seq-DGE	*WNT2*	RNA-ish showed heterogeneity of *WNT2* expression in CTCs and the primary tumor. This was confirmed by RNA-seq with DGE, showing rare *WNT2* RNA reads in the enriched CTC sample and the primary tumor.	37
*Melanoma*						
6	1	MagSweeper + MM	RNA-seq	Whole transcriptome	CTCs show a uniform upregulation of melanoma markers, including MAGE as well as uniform up- or downregulation of certain plasma membrane proteins.	128
*Multiple cancers*						
7, 29, 77	n.s.	^neg^CTC-iChip + MM	RNA-seq	Whole transcriptome	High expression of stromal-derived ECM proteins in > 15% of CTC samples. One glycoprotein was expressed in 100% of pancreatic CTCs compared to 31% of breast and 9% of prostate CTCs.	125

Abbreviations: CTC, circulating tumor cell; DGE, digital gene extraction; ECM, extracellular matrix; EMT, epithelial-to-mesenchymal transition; IM, immunomagnetic enrichment; MF, microfiltration; MM, micromanipulation; qRT-PCR, quantitative reverse transcription polyclonal chain reaction; RNA-ish, RNA *in situ* hybridization; RNA-seq, RNA sequencing; n.s., not specified.

### Metastasis-associated gene expression

In prostate cancer RT-PCR of 84 EMT-related genes was applied to analyze multiple single CTCs of 8 patients [129]. Heterogeneous upregulation of EMT-associated gene expression was found, especially in CRPC. RT-PCR was also used to target vimentin, EpCAM, and stem cell gene NANOG mRNA for EMT evaluation in approximately 400 breast CTCs [130]. Temporal heterogeneity was shown as expression patterns changed after surgery, with emerging of a sub-population of EpCAM positive CTC expressing NANOG and/or vimentin. Yu *et al.* applied RNA-ish for scoring the relative abundance of epithelial versus mesenchymal transcripts within individual breast cancer CTCs of 15 patients, both during therapy or at time of progression [81]. Clear heterogeneity was shown, with various proportions of CTCs that were mesenchymal. Moreover, relative changes during treatment in the expression of epithelial and mesenchymal markers in CTCs correlated with response and prognosis. For one patient, single CTCs were analyzed with RNA-ish over 7 time points and two different treatment regimens. An increased number of mesenchymal CTCs was repeatedly detected in the samples taken at time of disease progression [81]. Additionally, single molecule RNA-seq was applied on CTCs to identify signaling pathways that contribute to EMT, and 45 enriched genes were identified [81]. In metastatic pancreatic cancer, RNA-ish was used for detection of CTC-specific transcripts of Wnt2, which is known for its role in tumor sphere formation and metastasis initiation [37]. Wnt2 transcripts were identified in 23 out of 66 (35%) cytokeratin-positive CTCs from 2 out of 8 patients. Heterogeneity was also shown in the primary tumors. The small number of Wnt2-positive cells was consistent with RNA-seq analysis, which showed rare *Wnt2* RNA reads in both enriched CTCs and primary tumors [37]. This demonstrates Wnt2-positive CTCs are present at subclonal level and represent a rare subset of the primary tumor population. Ting and colleagues isolated 7, 29, and 77 single CTCs from patients with pancreas, breast, and prostate cancer respectively [125]. In more than 15% of all CTC samples, CTCs exhibit a very high expression of stromal-derived extracellular matrix (ECM) genes, which have an important role in metastatic spread. One specific ECM glycoprotein gene was expressed at high levels in 100% of pancreatic CTCs compared to 31% of breast and 9% of prostate CTCs [125].

### Global gene expression profiling

Recently, genome-wide expression profiling of single cells using NGS has been achieved [127, 128]. In a study regarding patients with metastatic breast cancer, a homogeneous global expression pattern was shown, with all CTCs clustering together patient wise, except for two patients [124]. Furthermore, in advanced melanoma, some highly expressed transcripts in single CTCs were detected [128]. Although slight differences in gene expression, CTCs show a uniform and high upregulation of cell-cycle and melanoma specific markers, as well as uniform up- or downregulation of certain plasma membrane proteins [128]. The same single cell mRNA-seq protocol was used for CTCs isolated from patients with metastatic prostate cancer [131]. High rates of RNA degradation consistent with apoptosis amongst CTCs was noted, although prostate-specific and cancer-specific transcripts could still be elucidated. 181 genes were overexpressed in the CTCs compared to normal prostate tissue [131]. Unsupervised clustering revealed that all CTCs, except two, cluster in a patient specific manner. Specific transcripts, e.g. related to CRPC or *ERG*-fusion, were detected homogeneously within the same patients [131]. In another RNA-seq study on prostate cancer, hierarchical clustering analysis also demonstrated patient-specific CTCs clustering, separated from cancer cell lines. However, single CTCs from nine individual patients with at least 3 CTCs analyzed, showed considerably higher heterogeneity in their transcriptional profiles compared to single cells from prostate cancer cell lines [126]. Moreover, RT-PCR of a panel of 87 cancer genes demonstrated heterogeneity among individual breast cancer CTCs, separating them into two major subgroups based on 31 highly expressed genes [132]. This was in contrast to several breast cancer cell lines tested.

### Prostate cancer specific gene expression

Isolated single CTCs were tested for expression level of the PSA gene *KLK3* [34]. The expression profile of *KLK3* was heterogeneous between the 26 out 48 selected individual CTCs, for which sufficient part of the transcriptome was covered [34]. Besides, expression patterns of AR splice variants have been studied at a single cell level using either RT-PCR or RNA-seq [73, 126]. One study demonstrated that more than half of all patients had multiple AR splice variants present within different CTCs and that a subpopulation of single CTCs had simultaneous expression of several AR splice variants [126]. These results are in line with other data showing that acquisition of AR-independent alterations conferring resistance to antiandrogen therapies is very heterogeneous in patients with CRPC [79]. Temporal heterogeneity between multiple enriched CTC samples from 21 patients with prostate cancer was shown by emerging of AR-V7 in one out nine patients treated with taxane chemotherapy. In contrast, seven out of twelve patients who were AR-V7 positive at baseline, only harbored full length AR at time of progression [73]. Relations between therapy response and presence of variants are increasingly studied [74, 97], although usually not at multiple time points or with multiple CTC samples, which is needed to study tumor evolution.

## DISCUSSION

### Technical considerations

Studies across multiple tumor types have demonstrated the feasibility of analyzing molecular profiles of single CTCs. Although technical improvements are needed, it becomes clear that CTC profiling contributes to our understanding of tumor heterogeneity, disease evolution (through serial sampling), and clinical management. To maximize the potential of CTC profiling, key issues in CTC research must be addressed regarding both technical and biological challenges.

Evolution in multiple-marker and marker-independent CTC enrichment has already increased yield and diversity of CTCs [50, 81, 133], although it is not as extensively validated as EpCAM enrichment strategies. Furthermore, efforts have been made to improve both amplification methods [134–136] and sequencing techniques [34, 127, 128] as well as subsequent data interpretation and bioinformatics [10, 137, 138], reviewed in more detail by Van Loo and Voet [24]. This all contributes to more reliable detection of aberrations and evaluation of heterogeneity in CTC research.

**Figure 2:**
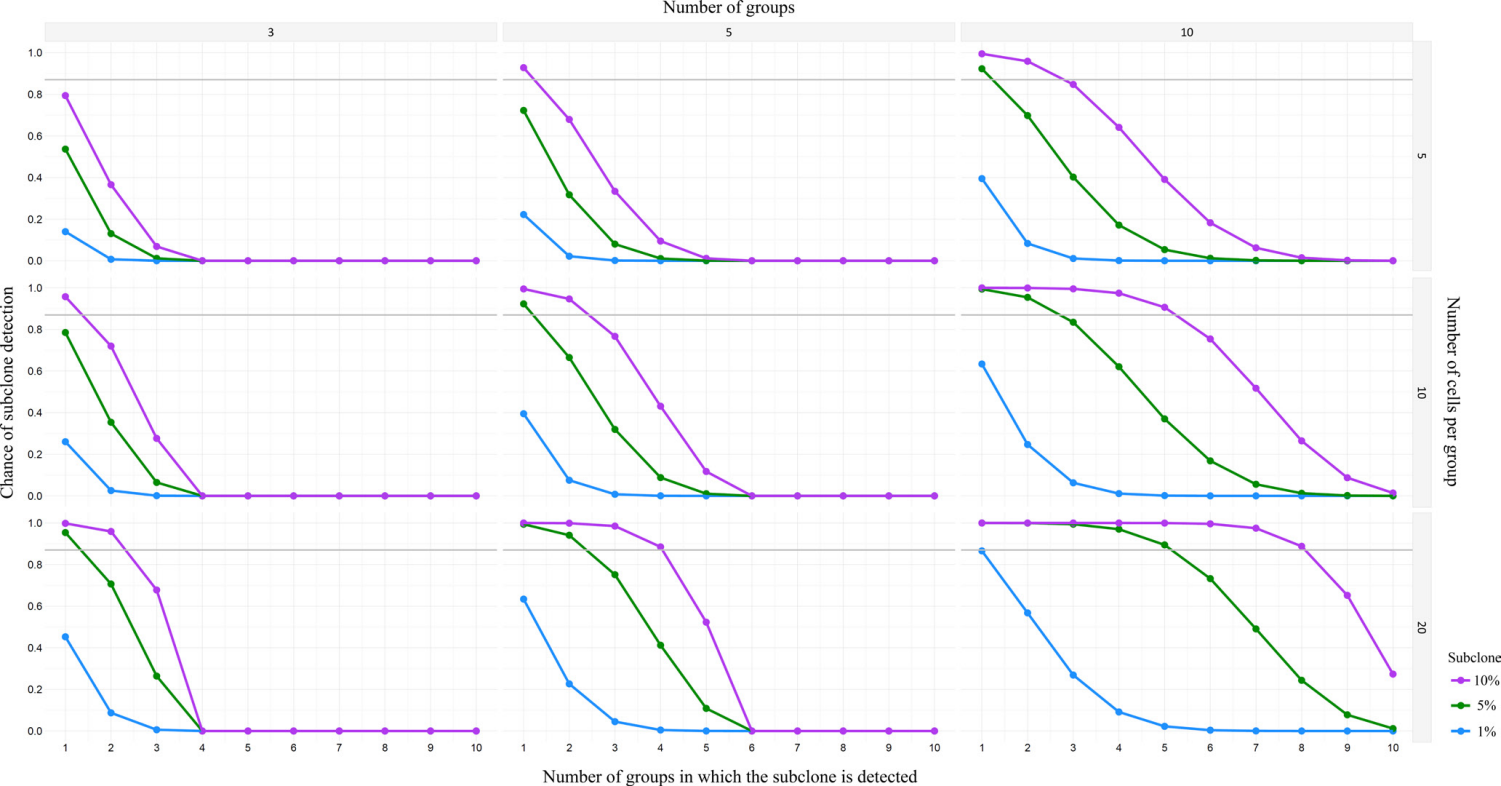
Power analysis for detection of minor subclones in pools of CTC Chances of detection of minor subclones (i.e. 1%, 5%, or 10%), calculated with a power of 0.87, for three different number of groups (i.e. 3, 5, or 10 groups) and three different number of cells per group (i.e. 5, 10, or 20 cells). As depicted in the lower right graph (10 groups of 20 cells), there is a 90% change of detecting a 1% subclone in 1 out of 10 groups, or detecting a 5% subclone in 5 out of 10 groups, or detecting a 10% subclone in 8 out of 10 groups.

A major question in CTC research remains how many CTCs should be profiled to account for heterogeneity. Often, the molecular characteristics of only a few CTCs out of the entire pool of CTCs from a patient have been adequately analyzed [34, 100]. As a consequence their diversity remains largely unknown. In primary breast cancer for example, single-molecule sequencing indicated that many of the diverse mutations occur at low frequencies (< 10%) in the tumor mass [139]. Navin demonstrated, using a power analysis, that detection of a 10% subclone would require sequencing at least 20 single cells to achieve a 0.87 detection power [140]. Besides, subclonality can be evaluated using multiple small pools of pure CTCs (Figure 2) and determining the variant allele frequencies. Herewith technical errors typical for single cell research [24] can be reduced, although more CTCs need to be available and isolated. Furthermore, in depth comparative research towards CTCs and multiple metastases [31] should clarify whether the whole tumor burden contributes equally to the CTC pool or if some subclones might be underrepresented or absent.

### Clinical implications and future perspectives

Currently, biomarkers predicting therapy response are frequently assessed using primary tumor biopsies, reflecting only parts of a patient's disease at a specific moment in time [141]. It is well-known that targetable molecules can change during the course of the disease. CTCs have shown to be useful in understanding and predicting acquired resistance to therapies, and might in the future be used to circumvent this. In lung cancer, serial analysis identified emergence of activating mutations in the *EFGR* gene in some patients receiving EGFR-targeting therapy, conferring a mechanism of acquired resistance to therapy [65]. Moreover, clonal selection of *ALK*-rearranged CTCs during crizotinib therapy was detected in patients with lung cancer [89]. Serial RNA analysis of prostate CTCs demonstrated emergence of AR-V7 during taxane chemotherapy [73], and TMPRSS2-ERG status in CTCs is a predictive biomarker of abiraterone acetate sensitivity in CRPC [97]. Hence, repeated CTC sampling may have the potential to guide optimal therapy regimens depending on the evolving molecular profile of the tumor burden within an individual patient. However, CTC characterization is currently only performed in clinical trials [142]. Therefore, efforts to increase clinical utility, have to be made. A comprehensive analysis of multiple patient samples, including CTCs, cfDNA, and tissue samples, on both RNA and DNA level can provide a holistic view of a patient's (sub)clonal landscape. The development of multi-compartment molecular databases of large patient cohorts will enable the creation of algorithms able to predict outcome at a more individual patient level [3, 143, 144].

A key issue remains to what extent heterogeneity in the circulating compartment affects therapy outcome and whether one should take a minor subclone into account if it comes to treatment selection. The analysis of subclonal heterogeneity may help clinicians understand why patients do not respond homogeneously to targeted drugs. Furthermore, longitudinal molecular analysis of individual CTCs can uncover clonal evolution caused by therapy pressure [32, 78, 145]. In a patient with CRPC, sequentially progressive on chemo and targeted therapy, comparable CTC clones were observed before the start and during standard chemotherapy. However, subsequent clinical response to targeted therapy was associated with the drastic depletion of the fist clone and emergence of a second clone, while a third tumor lineage was detected at time of disease progression [80]. As acquired drug resistance and disease relapse is common, drugs may only ablate specific subpopulations of tumor cells, allowing resistant cells to grow, evolve and seed new tumor foci that may not respond to cytotoxic or targeted therapies [32, 78, 145]. Hence, a tremendous potential of CTCs lies in profiling them over the entire clinical course to study the evolutionary history of tumors and to optimize clinical trial design. In the TRACERx trial (NCT01888601), primary tumors of 842 NSCLC patients will be sequenced, as well as cfDNA and CTCs, obtained at multiple time points during therapy. To evaluate the effect of clonal heterogeneity and selection pressure on clinical outcomes, and to identify targetable driver events, repeated tumor sampling will be performed at time of disease recurrence. These patients will be eligible for the DARWIN trial (NCT02183883). This trial aims at evaluating whether targeting driver events, detected by the TRACERx trial, has a different clinical outcome in patients harboring the driver dominantly compared to subclonally.

In conclusion, molecular characterization of CTCs provides the opportunity to repeatedly assess the biological features of cancer during the evolution of the disease. Therefore, CTCs may facilitate the development of new therapeutic strategies and enable clinicians to tailor therapy to an individual patient in a longitudinal fashion. The relevance of CTC heterogeneity as a cause or consequence of resistance to targeted therapy is yet to be unveiled. Hence, a tremendous potential of CTCs lies in single-cell profiling techniques that will contribute to understanding the predictive value of driver molecular aberrations in subclones of CTCs and emergence of resistant populations on targeted therapy.

## Abbreviations

General:

CGH: comparative genomic hybridization
CNA: copy number alteration
CRC: colorectal cancer
CRP: castration resistant prostate cancer
CTC: circulating tumor cell
DGE: digital gene extraction
EMT: epithelial-to-mesenchymal transition
FISH: fluorescent in situ hybridization
ITH: intra tumor heterogeneity
NCSLC: non-small cell lung cancer
NGS: next generation sequencing
SCS: single cell sequencing
SNV: single nucleotide variation
WGA: whole genome amplification
WGS: whole genome sequencing
WTA: whole transcriptome amplification

Genes:

*ALK*: anaplastic lymphoma kinase
*APC*: adenomatous polyposis coli
*BRAF*: v-Raf murine sarcoma viral oncogene homolog B
*BRCA1*: breast cancer 1
*CCND1*: cyclin-D1
*EGFR*: epidermal growth factor receptor
*EML4*: echinoderm microtubule-associated protein-like 4
*ERBB2*: avian erythroblastosis oncogene B 2
*ERG*: ETS-related gene
*KLK3*: kallikrein 3
*KRAS*: Kirsten rat sarcoma viral oncogene homolog
*MYC*: V-myc avian myelocytomatosis viral oncogene homolog
*PIK3CA*: phosphatidylinositol-4, 5-bisphosphate 3-kinase
*PTEN*: phosphatase and tensin homolog
*RB1*: retinoblastoma 1
*ROS1*: ROS Proto-Oncogene 1
*TMPRSS2*: transmembrane protease serine 2
*TP53*: tumor protein 53

Markers:

ALDH: aldehyde dehydrogenase
AR: androgen receptor
EpCAM: epithelial cell adhesion molecule
HER2: receptor tyrosine-protein kinase erbB-2
PSA: prostate-specific antigen
